# Curve Behavior of Distal Segments After Posterior‐Only Hemivertebra Resection for Congenital Cervicothoracic Scoliosis

**DOI:** 10.1111/os.14341

**Published:** 2024-12-23

**Authors:** Yang Li, Zezhang Zhu, Wanyou Liu, Saihu Mao, Zhen Liu, Xu Sun, Yong Qiu, Benlong Shi

**Affiliations:** ^1^ Division of Spine Surgery, Department of Orthopedic Surgery, Nanjing Drum Tower Hospital, Affiliated Hospital of Medical School Nanjing University Nanjing China

**Keywords:** cervicothoracic hemivertebra, compensatory type, distal curve progression, emerging scoliosis, hemivertebra resection, idiopathic type

## Abstract

**Study Design:**

A retrospective and consecutive study.

**Objective:**

To demonstrate the curve evolution of distal non‐structural compensatory curves in patients with congenital cervicothoracic hemivertebra (CTH) scoliosis undergoing posterior‐only hemivertebra resection and to propose the possible mechanisms of this specific phenomenon.

**Summary of Background Data:**

Though the spinal alignment could be well corrected via posterior hemivertebra resection in CTH patients, the high prevalence of distal curve progression was remarkable. However, the curve behavior of distal non‐structural compensatory curves and its possible mechanisms were unclear.

**Methods:**

This study retrospectively reviewed a consecutive series of CTH patients undergoing posterior‐only hemivertebra resection with a minimum 24 months follow‐up. The parameters measured in coronal plane included local scoliosis, clavicle angle, head shift, and the Cobb angle of distal unfused segments. The distal curve was considered as an emerging scoliosis (ES) if with more than 20° progression and the apex of distal curve no less than 2 levels away from the lower instrumented vertebra.

**Results:**

A total of 51 CTH patients with a mean age of 8.5 ± 3.8 years at surgery and a mean 38.0 ± 5.3 months follow‐up were recruited. The correction of local scoliosis and clavicle angle was statistically significant (*p* < 0.05 for all). The distal compensatory curve was 11.8 ± 5.3° before surgery and 6.5° ± 4.1° after surgery (*p* < 0.001), which was slightly increased to 11.6° ± 10.9° (*p* = 0.002) at the latest follow‐up. During follow‐up, the increase of distal compensatory curve was significantly correlated with the change in clavicle angle (*r* = 0.49, *p* = 0.038). The ES was observed in 10 patients (19.6%) with an average value of 28.0 ± 2.1° at diagnosis, including 7 patients within 6 months and 3 patients after 5 years postoperatively. The mean value of ES was 31.9° ± 3.1° at the latest follow‐up, while no patients required revision surgery. The ES was classified into compensatory and idiopathic types according to the typical curve behaviors. The compensatory ES usually presented within 6 months after operation and was responsible for further reconstruction of head and shoulder balance. While the idiopathic ES occurred at adolescent which may be related to the rapid body growth.

**Conclusions:**

Distal compensatory curve had a tendency toward slight progression during follow‐up in CTH patients with posterior hemivertebra resection surgery. The prevalence of emerging scoliosis was 19.6% and the typical compensatory and idiopathic curve behavior were firstly proposed. Close and longitudinal follow‐up was thus highly recommended for CTH patients with posterior HV resection surgery.

## Graphical abstract

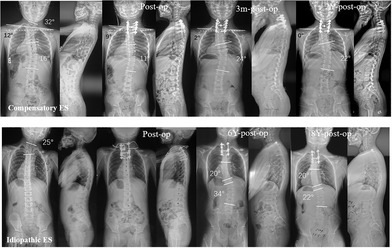

## 1 Introduction

Hemivertebra (HV) is the most frequent cause of congenital scoliosis (CS) which should usually be intervened at an early age [1, 2, 3]. Posterior HV resection with pedicle screw fixation has gradually become the mainstay treatment of this cohort, and satisfactory outcomes have been widely reported in the literatures [2, 4, 5]. The cervicothoracic hemivertebra (CTH), traditionally defined as HV located at the unique C6‐T4 regions, comprised a rare deformity in the CS cohort, and the surgical treatment of patients with CTH was significantly more difficult [6, 7]. Fortunately, previous studies have demonstrated that the spinal alignment could be well corrected via posterior HV resection with instrumentation in most patients with CTH [2, 4, 7, 8, 9]. However, along with the wide application of posterior HV resection, complications including implant failure, junction kyphosis, and coronal decompensation have been reported and addressed in detail [3, 5, 10]. Recently, the postoperative curve progression of distal unfused segments has drawn increasing attentions in CS patients with short fusion, which is believed to be more common in CTH patients [3, 5, 11, 12, 13, 14]. Huang et al. reported that the incidence of postoperative distal curve progression was 19% in CS patients with upper thoracic HV undergoing posterior HV resection [12]. Chen et al. reported 16 patients with a progressed distal compensatory cure in a consecutive series of 18 CTH patients with posterior‐only HV resection, among whom 4 (22%) patients had a distal curvature more than 20° at the last follow‐up.

In clinical practice, owing to the convoluted nature of spinal deformities in CTH patients, it was frequently arduous to make the anticipation of alterations in the distal unfused segments subsequent to hemivertebra resection and short‐segment fixation. Some patients might manifest substantial progression of the distal compensatory curvature after the surgical procedure. For patients exhibiting rapid progression of distal compensatory curvature that cannot be adequately managed by bracing, or those presenting with severe coronal imbalance, definitive revision surgery may be necessitated for effective intervention. Burkhardt et al. and Wang et al., respectively, chronicled 2 cases (11.8%, 2/17) and 3 cases (12%, 3/25) of patients with CTH who underwent revision surgery due to significant progression of distal unfused curvature subsequent to posterior HV resection and fusion [15, 16]. However, the detailed curve evolution of distal non‐structural compensatory curves in patients with CTH undergoing posterior HV resection during longitudinal follow‐up has not been well investigated.

In the current study, we retrospectively reviewed a series of patients with CTH undergoing posterior‐only HV resection with short fusion, aiming: (1) to demonstrate the curve evolution of distal non‐structural compensatory curves and (2) to propose the possible mechanisms of this specific phenomenon. We anticipate that our research will offer enhanced insights into the precise management and postoperative prognostic assessment of patients with CTH.

## 2 Materials and Methods

### 2.1 Subjects

This study was approved by the Ethics Committee of our Hospital (Approval No.: 2021⁃398⁃01). Our indications for surgical intervention were those patients with a fully segmented or nonincarcerated hemivertebra, which was considered to be poor prognosis. Patients with CTH undergoing one‐stage posterior‐only HV resection and fusion surgery between June 2005 and January 2020 were retrospectively reviewed.

The inclusion criteria were: (1) HV located from C6 to T4; (2) with a relative short fusion segments (≤ 8 levels) and the lower instrument vertebra (LIV) was above T8; (3) with a minimum of 24 months follow‐up. Exclusion criteria were as follows: (1) with structure secondary curve before surgery which was defined as more than 10° on bending films [11]; (2) concomitant HV or other vertebral abnormalities located at other spinal regions; (3) with arthropathy or discrepancy of lower limbs. Patients were requested to finish the Scoliosis Research Society‐22 (SRS‐22) questionnaire.

### 2.2 Surgical Strategy

Correction procedures were generally performed in the following order: (1) insertion of pedicle screws or hooks; (2) HV resection; (3) bone to bone fusion and instrumentation; (4) T1 horizontalization. Resection of main HV that gave rise to the scoliosis was performed via posterior‐only approach [17]. The selection of fusion levels was basically HV ± 2, while the fusion levels should be prolonged for those with poor pullout strength of screws or pronounced kyphoscoliosis [7]. The ideal coronal and sagittal balance was the main goal of the correction surgery, and minimal segment fusion was the priority to preserve more growth potential.

Standing spinal coronal and lateral radiographs were evaluated preoperatively and postoperatively. The following parameters were measured: (1) Cobb angles of local scoliosis and the curve of distal unfused segments. The distal curve was considered as an emerging scoliosis (ES) if with more than 20° increase than immediate postoperation and the apical vertebra was at least 2 levels from the LIV [11]; (2) T1 tilt, the angle between the horizontal line and line through the upper endplate of T1; (3) clavicle angle, the angle between the horizontal line and the tangential line connecting the highest two points of each clavicle; (4) neck tilt, the angle between the longitudinal axis of the cervical spine (the line drawn from center of the C2 odontoid process and the center of C7) and the vertical line; and (5) head shift, the distance between a vertical line drawn from the middle of the mandibular body to the middle of the sacrum [7]. In addition, the segmental kyphosis, thoracic kyphosis, and lumbar lordosis were measured.

### 2.3 Statistical Analysis

SPSS software 17.0 (SPSS Inc., Chicago, IL) was used for statistical analysis. A paired‐sample *t*‐test was conducted to assess the differences between preoperative and postoperative measurements. The Pearson coefficient was applied for the correlation analysis. Employing the occurrence of ES as the dependent variable (where the presence of ES was designated as 1 and its absence as 0), and taking the discrepant factors with statistical significance in the univariate analysis as independent variables, a binary logistic regression analysis was performed to clarify the risk factors for the progression of distal non‐structural compensatory curve. The statistical significance was set as *p* < 0.05.

## 3 Results

A total of 51 patients, with a mean age of 8.5 ± 3.8 years (range: 3.8 to 14 years), were ultimately included in this study, following the loss to follow‐up of eight patients. The mean duration of postoperative follow‐up was 38.0 ± 5.3 months. There were 22 (43%) patients with single HV, 19 patients (37%) with double HVs, and 10 patients (20%) with multi‐level HVs. The mean fusion segments were 5.9 ± 1.2 levels. The correction of local scoliosis, shoulder balance, T1 tilt, and clavicle angle was statistically significant (*p* < 0.05 for all), and the detailed data were shown in Table 1. No significant alterations were observed in segmental kyphosis, thoracic kyphosis, or lumbar lordosis across the preoperative, postoperative, and final follow‐up assessments (*p* > 0.05 for all). The average distal compensatory curve was 11.8° ± 5.3° before surgery and 6.5° ± 4.1° after surgery (*p* < 0.001), which increased to 11.6° ± 10.9° at the last follow‐up. During follow‐up, the increase of distal compensatory curve was significantly correlated with the change in clavicle angle (*r* = 0.49, *p* = 0.038).

**TABLE 1:** Radiographic parameters of preoperative, postoperatively, and last follow‐up.

	Preoperative	Postoperative	Last follow‐up	*p* ^a^	*p* ^b^
Local scoliosis	42.9 ± 4.8	18.3.5 ± 3.2	20.1 ± 5.8	< 0.001	0.056
T1 tilt	17.9 ± 6.1	10.2 ± 4.6	7.7 ± 4.4	< 0.001	0.006
Clavicle angle	16.2 ± 7.1	8.8 ± 6.9	5.2 ± 5.1	< 0.001	0.003
Neck tilt	21.9 ± 8.0	12.9 ± 7.3	10.8 ± 6.9	< 0.001	0.139
Head shift	23.3 ± 10.5	18.8 ± 10.3	16.4 ± 9.8	0.031	0.231
Distal compensatory curve	11.8 ± 5.3	6.5 ± 4.1	11.6 ± 10.9	< 0.001	0.002
Segmental kyphosis	15.8 ± 5.0	14.3 ± 6.2	14.9 ± 4.9	0.182	0.589
Thoracic kyphosis	28.6 ± 10.1	29.8 ± 9.1	29.9 ± 9.3	0.529	0.956
Lumbar lordosis	33.3 ± 11.7	32.9 ± 10.5	34.4 ± 12.3	0.856	0.509

^a^ Comparison between preoperative and postoperative indicators.

^b^ Comparison between the postoperative and last follow‐up indicators.

Horner syndrome occurred in 3 patients after surgery, of whom all symptoms were relieved within six months after surgery. Leakage of cerebrospinal fluid occurred in 4 patients without severe clinical symptoms.

Infection, pseudoarthrosis, and implant failure was not noted during the follow‐up.

Notably, 10 patients (19.6%) developed an ES during the follow‐up period, with a mean age at surgery of 7.5 ± 2.1 years. The mean values for ES were recorded as 28.0° ± 2.1° at the time of diagnosis and increased to 31.9° ± 3.0° at the last follow‐up (*p* < 0.001). Five patients with ES were treated with brace for an average of 16 h per day, which was improved from 31.0° ± 2.3° at the time of diagnosis to 22.4° ± 2.1° at last follow‐up (*p* < 0.001). whereas the remaining 5 patients underwent closely observation. At the last follow‐up, no revision surgery was performed. (Table 2) At the final follow‐up, the total SRS‐22 score was significantly higher in patients without ES (4.5 ± 0.4) compared to those with ES (4.1 ± 0.3) (*p* = 0.005). While function and pain scores were comparable between both groups, the other three items in the ES group were notably lower than those in the non‐ES group at last follow‐up (*p* < 0.05) (Table 3). We then categorized the ES into compensatory and idiopathic types according to the typical curve behaviors, which will be elaborated upon meticulously hereinafter: (1) Compensatory ES, which typically manifests within 6 months postoperatively with a curvature direction consistent with the preoperative distal compensatory curve; (2) Idiopathic ES, which emerges more than 6 months postoperatively characterized by a de novo distal curve.

**TABLE 2:** Patient's demographic and radiographic characteristics.

Case	Sex	Age (y)	Location of HV (resected HV)	Compensatory curve			Fused segments	ES			Treatment
				Levels	Preoperative Cobb angle (°)	Postoperative Cobb angle (°)		Onset (months after operation)	Levels	Cobb angle at diagnose (°)	
1	M	7	C7, T2 (C7)	T7‐L1	14	12°	C6‐T3	3	T9‐L1	24	Observation
2	F	9	T3 (T3)	T6‐L3	8	2°	T1‐T5	6	T6‐L4	24	Observation
3	M	10	C7, T2, T3 (T2)	—	—	—	C6‐T6	6	T8‐L2	29	Brace
4	F	6	T1 (T1)	—	—	—	C7‐T3	3	T9‐L2	30	Brace
5	M	4	T1, T2, T3 (T3)	T5‐L3	12	6°	C7‐T7	6	T6‐L3	28	Observation
6	F	5.5	T2 (T2)	—	—	—	T1‐T5	3	T6‐L4	29	Brace
7	F	7	T3 (T3)	T4‐L1	10	3°	T1‐T6	3	T4‐L1	26	Observation
8	F	11	T3 (T3)	T11‐L2	11	8°	C7‐T6	66	T7‐L1, L1‐L5	18, 33	Brace
9	F	8	T2, T4 (T4)	T10‐L3	10	6°	T1‐T7	48	T12‐L4	29	Observation
10	M	7	T2, T4 (T2)	—	—	—	C7‐T6	72	T8‐T12, 12‐L5	20, 34	Brace

**TABLE 3:** The SRS‐22 score preoperatively and at last follow‐up.

	Preoperative	Last follow‐up	*p* ^a^	*p* ^b^
Function				
ES group	4.2 ± 0.4	4.3 ± 0.5	0.320	0.503
No ES group	4.3 ± 0.6	4.4 ± 0.4	0.666	
Pain				
ES group	4.4 ± 0.6	4.3 ± 0.7	0.695	0.603
No ES group	4.3 ± 0.7	4.4 ± 0.5	0.717	
Self‐image				
ES group	3.1 ± 0.5	4.2 ± 0.4	< 0.001	< 0.001
No ES group	3.3 ± 0.4	4.8 ± 0.5	< 0.001	
Mental health				
ES group	3.4 ± 0.4	4.0 ± 0.3	< 0.001	< 0.001
No ES group	3.6 ± 0.3	4.5 ± 0.4	< 0.001	
Satisfaction				
ES group	—	3.9 ± 0.6	—	< 0.001
No ES group	—	4.5 ± 0.4	—	
Total				
ES group	3.8 ± 0.4	4.1 ± 0.3	< 0.001	0.005
No ES group	3.9 ± 0.3	4.5 ± 0.4	0.001	

^a^ Comparison between preoperative and postoperative indicators.

^b^ Comparison between the indicators of two groups at last follow‐up.

**FIGURE 1:**
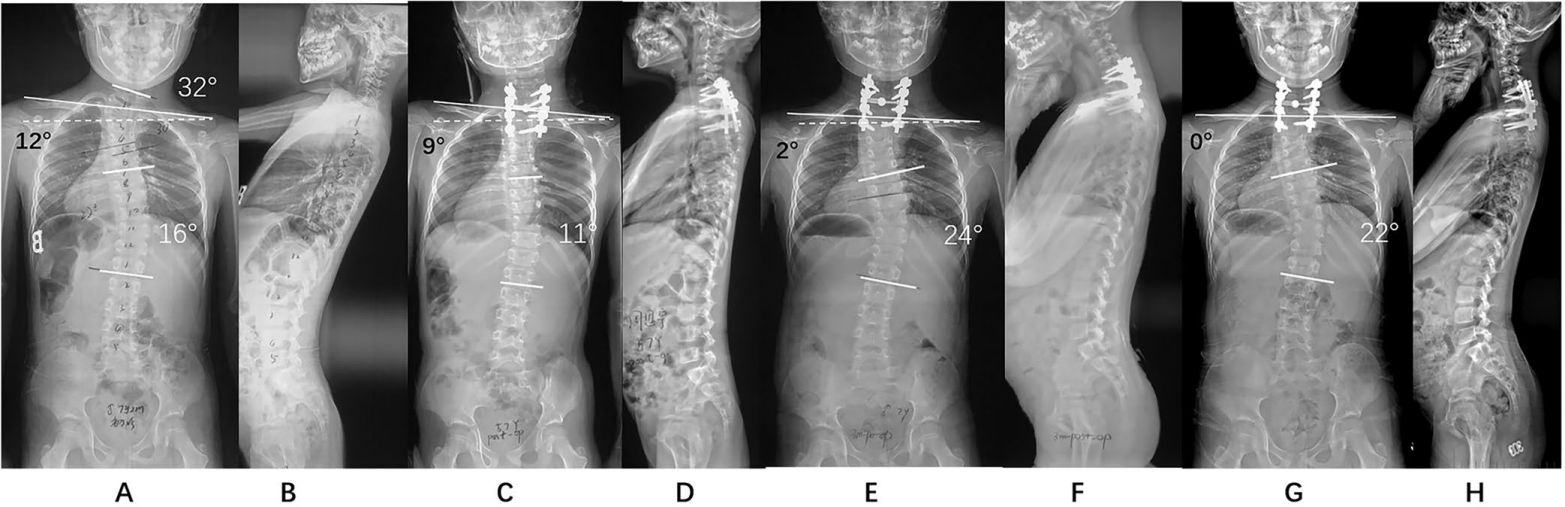
A demo case of compensatory ES type. The patient was a 7 years old boy with C7 and T2 HVs. The local scoliosis was 32° with a distal compensatory curve of 16° and a clavicle angle of 12° (A & B). The patient underwent C7 HV resection and C6 to T3 fusion. The postoperative clavicle angle was 9°, and the local scoliosis and head shift were well corrected. Distal compensatory curve was spontaneously corrected to 11° after surgery (C & D). The distal curve progressed to 24° at 3 months after surgery (E & F), and was consistant at 2 years follow‐up (G & H).

The ES occurred within 6 months after surgery in 7 patients (70%), including 4 patients at 3 months and 3 patients at 6 months postoperatively. The ES of these patients was characterized by a thoracolumbar curve, with the same curve direction and region as preoperative distal compensatory curve. All 7 patients exhibited postoperative shoulder imbalance, characterized by a higher postoperative clavicle angle of 12.4° ± 2.9° compared to the overall average indicator of 8.8° ± 6.9°, although this difference was not statistically significant (*p* = 0.181). At the last follow‐up, however, the clavicle angle decreased to 5.5° ± 1.1°, accompanied by an increase in the compensatory curve. When a binary logistic regression analysis was conducted with the appearance of ES as the dependent variable (appearance of ES = 1, absence = 0), it was found that residual postoperative clavicle angle was a risk factor for the appearance of ES (OR = 1.109, 95% CI [0.881, 1.237], *p* = 0.048). The ES thus served as an important compensatory mechanism during the reconstitution of shoulder balance, which was defined as “compensatory ES” (Figure 1).

**FIGURE 2:**
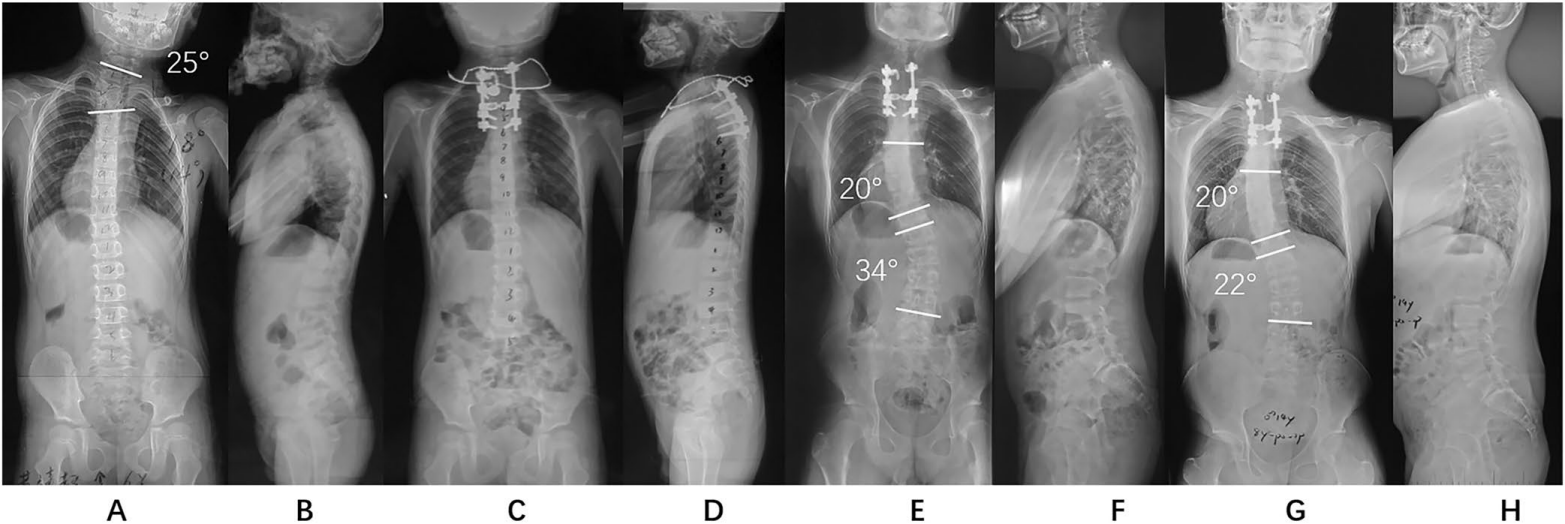
A demo case of idiopathic ES type. The patient was a 7 years old boy with a local scoliosis of 25° (A & B). The patient underwent T2 HV resection and C7 to T6 fusion. The local scoliosis, head shift, and shouder balance were corrected to 8°, 7.1 mm, and 1°, respectively, after surgery (C & D). ES was observed at 6 years follow‐up with an “idiopathic‐like” ES and brace treatment was proposed (E & F). The distal curve was improved to 22° at 8 years follow‐up (G & H).

Three patients (30%) presented a classical S‐shaped ES at 5.5, 4, and 6 years after surgery. The postoperative clavicle angle was 4°, 5°, and 3° respectively. The postoperative neck tilt was 8°, 7°, and 6° respectively. The local scoliosis was 11°, 13°, and 9°, respectively. The data evinced that all three patients attained rather satisfying deformity correction operation. No manifestations of distal ES were discerned during the short‐term follow‐up. Nevertheless, astonishingly, all three patients manifested ES during the long‐term follow‐up. Additionally, the characteristic of ES displayed by these three patients were discordant with those of “compensatory ES” in terms of both onset chronology and curvature attributes; accordingly, we posit that the mechanisms underpinning this type of ES constitute a sui generis category. The ES occurred at puberty and rapidly progressed during growth spurt, of which the curvature feature bears a striking resemblance to idiopathic scoliosis. Thereby, we defined this type of ES as an “idiopathic ES”. Hence, we inferred that rapid growth of the body may be responsible for this complication (Figure 2).

## 4 Discussion

The hemivertebra resection with short segmental fusion via posterior‐only approach at an early stage was confirmed as an effective strategy with satisfactory radiographic and clinical outcomes. However, the distal curve progression was found to be remarkable during follow‐up in patients with CTH who underwent hemivertebra resection and fusion. This study demonstrated the curve evolution of distal non‐structural compensatory curves in patients with CTH patients undergoing posterior‐only hemivertebra resection and proposed the possible mechanisms of this specific phenomenon, which we believe could provide more evidence for the precise management and postoperative prognostic assessment of patients with CTH.

### 4.1 Distal Curve Progression Was a Critical Compensatory Mechanism in CTH Patients

The goal for surgical treatment of CS was to achieve a physiologically rebalanced spine with minimal solid fusion [2, 5]. To fulfill the demanding goals, early diagnosis and appropriate surgical strategy were crucial. The nature and history of CS demonstrated a high risk of rapid curve progression, during which both the primary and compensatory curves could progress to be rigid and structural [18, 19]. The HV resection with short segmental fusion via posterior‐only approach at an early stage was confirmed as an effective strategy with satisfactory radiographic and clinical outcomes [7]. However, there were only a few studies focusing on patients with CTH who often presented with prominent deformities such as should unbalance, neck tilt, head shift, and others. Since the cervicothoracic junction severs as a transitional region between the highly flexible cervical spine and the rigid thoracic spine, more attentions on postoperative complications, especially the distal curve progression, [11, 12] had been paid recently to CTH patients with posterior‐only HV resection and fusion.

Chen et al. [7] demonstrated a tendency toward slight progression of distal compensatory curve during follow‐up in CTH patients undergoing posterior HV resection. Their results revealed a positive correction between distal compensatory curve change and further improvement of head shift and clavicle angle, [7] which was in accordance with our results. With slight progression of the distal compensatory curve, the clavicle angle, which was correlated with shoulder balance, decreased consistently to restore shoulder balance in a better status [20, 21]. Additionally, neck tilt and head shift consistently decreased during the follow‐up period, although no significant differences were observed when compared to postoperative measurements. Therefore, we can infer that the distal unfused segments play a critical role in the further reconstruction of head and shoulder balance through distal curve progression.

### 4.2 The Incidence of ES in CTH After Surgery

Although the distal curve progression functioned as a critical compensatory mechanism, the ES however was still an unexpected complication in this cohort. Previous studies have reported this rare complication in CS patients with thoracic or thoracolumbar HV. Yang et al. revealed 9 (7.0%) of 128 CS patients undergoing posterior HV resection with a postoperative‐emerging scoliosis more than 20°, of whom revision surgery was performed in 4 patients [11]. Similarly, Li et al. [3] reported the incidence of postoperative‐emerging curve was 10% in 179 thoracolumbar HV patients. The current study further proved that the incidence of ES was up to 19.6% in CTH patients undergoing posterior HV resection. In addition to the results of Chen et al. [7] in 2017 and Huang et al. [12] in 2019, we thereby believed that ES was notably more prevalent in CTH patients.

### 4.3 The Mechanisms of Postoperative ES in CTH Patients

Though postoperative progressive deformities in CS patients have been reported grandly, [22, 23, 24] the causes of ES were still ambiguous. It had a significant impact on the enhancement of patients' SRS‐22 scores, particularly regarding their self‐image and satisfaction. To optimize postoperative correction outcomes, it is essential to investigate potential factors influencing ES. Huang et al. [12] proposed that morphology change of paravertebral muscle and facet joint during operation should be responsible for the occurrence of ES. Li et al. [3] identified that higher preoperative LIV translation was an independent risk factor for ES in young CS patients with thoracolumbar HV. However, LIV was confined to upper thoracic vertebrae in CTH patients which was far away from distal curves. Hypothesis of morphology change in paravertebral muscle or LIV translation leading to ES may be inapplicable in CTH patients. Previous study demonstrated the compensatory rule of the increased distal compensatory curve in the further correction of head and shoulder balance in CTH patients [7]. In this cohort, the location of ES had a higher consistency with preoperative compensatory curve, highly implying the reasonability of this hypothesis. However, increasing evidences implied that the compensatory theory was only one of the mechanisms of ES in CTH patients. According to our clinical practice, we observed that the head and shoulder balance was well reconstructed in certain CTH patients undergoing HV resection. While, an idiopathic ES was observed unexpectedly during the longitudinal postoperative follow‐up. The progressive distal curve in these patients had no significant correlation with the cephalic HV resection surgery. The most significant characteristics of these ES was its occurrence at adolescence and the second growth‐peak. Besides, the curve evolution of “idiopathic‐like” ES were quite similar to the curves in patients with adolescent idiopathic scoliosis. Hence, we inferred that rapid growth of the body may be responsible for this complication. Fortunately, despite the high incidence of ES in CTH patients with posterior‐only HV resection and short fusion, the majority of distal curve progression could be controlled with the treatment of bracing or observation. Therefore, longer observation and follow‐up should be recommended to post‐operative CTH patients and their parents.

## 5 Limitations

The limitation of this study should be noted. First, loss of follow‐up was an inevitable short‐come in retrospective study. Thus, the real incidence of ES may be underestimated. Second, the follow‐up period was still relatively short in the majority patients and the curve evolution of the two types of ES during longitudinal follow‐up need to be further observed. Third, the curve pattern and fusion strategy of the patients included were various. Last, the sample size of this study was limited, which may introduce bias into the conclusions. Therefore, further confirmation from a larger number of cases was necessary.

## 6 Conclusions

This study firstly focused on the distal curve progression in CTH patients undergoing posterior HV resection and short fusion, of whom the prevalence of ES was 19.6%. The ES could be classified into compensatory and idiopathic types, which had distinct curve behavior during follow‐up. The compensatory ES usually presented within 6 months after operation and was responsible for further reconstruction of head and shoulder balance. While the idiopathic ES occurred at adolescent which may be related to the rapid body growth. Therefore, careful observation during longitudinal follow‐up was highly recommended to CTH patients undergoing posterior HV resection.

## Author Contributions

**Yang Li:** the conception and design of the study; analysis and interpretation of data; drafting the article; revising it critically for important intellectual content; final approval of the version to be submitted. **Zezhang Zhu:** provided administrative and intellectual support. **Wanyou Liu:** acquisition of data; analysis and interpretation of data; **Saihu Mao:** acquisition of data; analysis and interpretation of data; **Zhen Liu:** acquisition of data; analysis and interpretation of data; **Xu Sun:** acquisition of data; analysis and interpretation of data; **Yong Qiu:** the conception and design of the study; acquisition of data. **Benlong Shi:** the conception and design of the study; revising the article critically for important intellectual content; finalized and responsible for the manuscript. All authors read and approved the final manuscript.

## Conflicts of Interest

The authors declare no conflicts of interest.

## References

[1] M. J. McMaster and K. Ohtsuka , “The Natural History of Congenital Scoliosis. A Study of Two Hundred and Fifty‐One Patients,” Journal of Bone and Joint Surgery (American Volume) 64 (1982): 1128–1147. 7130226

[2] B. Yaszay , M. O'Brien , H. L. Shufflebarger , et al., “Efficacy of Hemivertebra Resection for Congenital Scoliosis: A Multicenter Retrospective Comparison of Three Surgical Techniques,” Spine (Phila pa 1976) 36 (2011): 2052–2060. 22048650 10.1097/BRS.0b013e318233f4bb

[3] S. Li , Z. H. Chen , Y. Qiu , et al., “Coronal Decompensation After Posterior‐Only Thoracolumbar Hemivertebra Resection and Short Fusion in Young Children With Congenital Scoliosis,” Spine 1 (2017): 654–660. 10.1097/BRS.0000000000002383 28816828

[4] Q. Zhuang , J. Zhang , S. Li , S. Wang , J. Guo , and G. Qiu , “One‐Stage Posterior‐Only Lumbosacral Hemivertebra Resection With Short Segmental Fusion: A More Than 2‐Year Follow‐Up,” European Spine Journal 25 (2016): 1567–1574. 26006704 10.1007/s00586-015-3995-x

[5] J. Guo , J. Zhang , S. Wang , et al., “Surgical Outcomes and Complications of Posterior Hemivertebra Resection in Children Younger than 5 Years Old,” Journal of Orthopaedic Surgery and Research 11 (2016): 48. 27113726 10.1186/s13018-016-0381-2 PMC4845311

[6] M. D. Smith , “Congenital Scoliosis of the Cervical or Cervicothoracic Spine,” Orthopedic Clinics of North America 25 (1994): 301–310. 8159403

[7] Z. Chen , Y. Qiu , Z. Zhu , et al., “Posterior‐Only Hemivertebra Resection for Congenital Cervicothoracic Scoliosis: Correcting Neck Tilt and Balancing the Shoulders,” Spine 43 (2017): 1. 10.1097/BRS.0000000000002325 28700454

[8] S. Cao , X. Chen , S. Pan , et al., “Evaluation and Comparation of a Novel Surgical Technique and Hemivertebra Resection to the Correction of Congenital Cervical Scoliosis in Lower Cervical and Cervicothoracic Spine,” Neurospine 19, no. 4 (2022): 1071–1083. 36397249 10.14245/ns.2244554.277 PMC9816581

[9] H. Q. Zhang , Y. X. Du , J. Y. Liu , et al., “Strategy and Efficacy of Surgery for Congenital Cervicothoracic Scoliosis With or Without Hemivertebra Osteotomy,” Orthopaedic Surgery 14, no. 9 (2022): 2050–2058. 36040110 10.1111/os.13480 PMC9483056

[10] X. Chen , L. Xu , Y. Qiu , et al., “Incidence, Risk Factors, and Evolution of Proximal Junctional Kyphosis After Posterior Hemivertebra Resection and Short Fusion in Young Children With Congenital Scoliosis,” Spine (Phila Pa 1976) 43 (2018): 1193–1200. 29419719 10.1097/BRS.0000000000002593

[11] X. Yang , Y. Song , L. Liu , et al., “Emerging S‐Shaped Curves in Congenital Scoliosis After Hemivertebra Resection and Short Segmental Fusion,” Spine Journal 16 (2016): 1214–1220. 10.1016/j.spinee.2016.06.006 27343728

[12] Y. Huang , G. Feng , L. Liu , et al., “Posterior Hemivertebral Resection for Upper Thoracic Congenital Scoliosis: Be Aware of High Risk of Complications,” Journal of Pediatric Orthopaedics. Part B 28 (2019): 1–9. 30308554 10.1097/BPB.0000000000000538

[13] Z. Liu , B. Jiang , Y. Jiang , et al., “Progressive Coronal Caudal Curve After Corrective Osteotomies for Congenital Cervicothoracic Scoliosis: Incidence and Predictors,” European Spine Journal 33, no. 4 (2024): 1675–1682. 38459986 10.1007/s00586-024-08189-7

[14] K. Sun , X. Sun , Z. Zhu , et al., “A Novel Classification of Congenital Cervicothoracic Scoliosis: Identification of Coronal Subtypes and Their Prognostic Significance,” European Spine Journal 33, no. 12 (2024): 4426–4436. 39443372 10.1007/s00586-024-08527-9

[15] B. W. Burkhardt , C. Meyer , G. Wagenpfeil , T. R. Pitzen , and M. Ruf , “The Effect of Cervicodorsal Hemivertebra Resection on Head Tilt and Trunk Shift in Children With Congenital Scoliosis,” Journal of Pediatric Orthopedics 40 (2020): e256. 31923019 10.1097/BPO.0000000000001506

[16] S. Wang , G. Lin , Y. Yang , et al., “Outcomes of 360° Osteotomy in the Cervicothoracic Spine (C7‐T1) for Congenital Cervicothoracic Kyphoscoliosis in Children,” Journal of Bone and Joint Surgery. American Volume 101 (2019): 1357–1365. 31393426 10.2106/JBJS.18.01428

[17] Y. Zhang , J. Yang , L. Zhou , A. Pan , and Y. Hai , “Selective Hemivertebrae Resection in a Congenital Scoliosis Patient With Multiple Hemivertebrae Deformities,” European Spine Journal 26 (2017): 1577–1583. 28281004 10.1007/s00586-017-4960-7

[18] M. Ruf and J. Harms , “Hemivertebra Resection by a Posterior Approach: Innovative Operative Technique and First Results,” Spine (Phila Pa 1976) 27 (2002): 1116–1123. 12004182 10.1097/00007632-200205150-00020

[19] M. Ruf and J. Harms , “Pedicle Screws in 1‐ and 2‐Year‐Old Children: Technique, Complications, and Effect on Further Growth,” Spine (Phila Pa 1976) 27 (2002): E460–E466. 12438997 10.1097/00007632-200211010-00019

[20] X. S. Qiu , W. W. Ma , W. G. Li , et al., “Discrepancy Between Radiographic Shoulder Balance and Cosmetic Shoulder Balance in Adolescent Idiopathic Scoliosis Patients With Double Thoracic Curve,” European Spine Journal 18 (2009): 45–51. 19043746 10.1007/s00586-008-0833-4 PMC2615117

[21] Y. Atici , Y. E. Akman , M. B. Balioglu , and S. Erdogan , “A Comparison of the Effects of Two Different Techniques on Shoulder Balance in the Treatment of Congenital Scoliosis: Vertical Expandable Prosthetic Titanium Rib and Dual Growing Rod,” Journal of Craniovertebral Junction and Spine 6 (2015): 190–194. 26692697 10.4103/0974-8237.167880 PMC4660496

[22] Y. Wang , N. Kawakami , T. Tsuji , et al., “Proximal Junctional Kyphosis Following Posterior Hemivertebra Resection and Short Fusion in Children Younger Than 10 Years,” Clinical Spine Surgery 30 (2017): E370–E376. 28437340 10.1097/BSD.0000000000000245

[23] J. Zhang , W. Shengru , G. Qiu , B. Yu , W. Yipeng , and K. D. K. Luk , “The Efficacy and Complications of Posterior Hemivertebra Resection,” European Spine Journal 20 (2011): 1692–1702. 21318279 10.1007/s00586-011-1710-0 PMC3175869

[24] Z. Shi , Q. Li , B. Cai , et al., “Causes of the Failure and the Revision Methods for Congenital Scoliosis due to Hemivertebra,” Congenit Anom (Kyoto) 55 (2015): 150–154. 25711333 10.1111/cga.12107

